# Unmet needs for proactive health services among older adults with functional impairment in Chinese long-term care facilities (LTCFs): a qualitative study

**DOI:** 10.3389/fpubh.2026.1861499

**Published:** 2026-08-14

**Authors:** Yixin Wang, Lanyue Luo, Liyu Chen, Hong Zhang, Xue Xiong, Dan Huo, Yuan Jia, Jun Shen

**Affiliations:** 1 Department of Nursing, The First Affiliated Hospital of Chongqing Medical University, Chongqing, China; 2 Chongqing Nursing Vocational College, Chongqing, China

**Keywords:** long-term care facilities, older adults with functional impairment, proactive health, qualitative research, unmet needs

## Abstract

**Objective:**

Proactive health, as an important strategy for addressing population aging in China, has not yet developed into a systematic service model for the care of older adults with functional impairment in LTCFs. This study aimed to systematically explore the unmet needs of proactive health services among older adults with functional impairment in LTCFs through a qualitative approach, and to provide a theoretical basis for the construction of a capability-based care system.

**Methods:**

A combination of purposive and maximum variation sampling was used to recruit 15 older adults with functional impairment (mild to severe) and 13 frontline nursing staff from an LTCF in Chongqing for semi-structured interviews conducted between July and September 2025. The interview data were analyzed using qualitative content analysis.

**Results:**

Four interrelated main themes were identified, covering individual capacity, psychosocial support, and systemic resource dimensions: autonomy and health decision-making involvement, health literacy enhancement and cognitive empowerment, psychological motivation and social connectedness, and structural health promotion resources and service systems.

**Conclusion:**

These unmet needs collectively hinder the transition from passive compensatory care to proactive health management. Meeting the proactive health needs of older adults with functional impairment in LTCFs requires the construction of a three-dimensional empowerment system of “individual-institution-society”: restoring autonomy through decision-making empowerment, enhancing cognitive capacity through tiered health education, strengthening psychological connection through social network reconstruction, and integrating service resources through mechanisms for coordinating medical and older adult care.

## 1 Introduction

Population aging has become one of the major challenges in global public health in the 21st century. According to the *United Nations’ World Population Prospects 2024*, the number of people aged 65 years and above is projected to exceed the number of those under 18 for the first time by the end of the 2070s (1). China is one of the countries experiencing the fastest population aging in the world. The proportion of people aged 60 years and above in China increased from 12.4% in 2010 to 22% by the end of 2025, reaching 310.31 million (2). This trend has been accompanied by a sharp increase in the number of older adults with functional impairment, which has reached approximately 35 million and accounts for 11.6% of the total older population (3). In LTCFs, this group accounts for 67% of residents and constitutes a core population served by these institutions (4). As they typically face multiple challenges, including declining physical function, chronic disease management, psychological adaptation, and limited social participation, their care needs often extend beyond basic assistance with daily living (5). However, only 49.3% of institutions can provide integrated closed-loop health management services covering physical, psychological, and social dimensions (6). This suggests that many LTCFs remain largely centered on basic survival over functional maintenance. Older adults may, to some extent, continue to receive care in a largely passive manner.

One important reason for this supply–demand gap is that the service model of long-term care facilities has not yet fully shifted from passive compensatory care toward a health promotion-oriented approach. In its *2015 World Report on Aging and Health*, the World Health Organization (WHO) stated that institutional care should shift from merely providing “compensatory services” to creating a supportive “health-promoting environment.” It also emphasized the need to maintain older adults’ functional independence through supportive strategies (7). However, in many Chinese LTCFs, this transition remains incomplete. Although basic physiological needs are usually met, fixed daily routines, uniform meal arrangements, staff-led activity choices, and safety-based restrictions may limit opportunities for older adults with functional impairment to make choices and use their remaining abilities. Over time, this may weaken their sense of autonomy and self-efficacy in daily life, increase their reliance on care, and contribute to functional decline and loss of dignity (8, 9). Therefore, promoting functional maintenance and health participation while meeting basic care needs has become an important direction for the development of long-term care services.

Some countries have developed specific models in line with this direction. In the United States, the Function Focused Care program emphasizes the use of remaining functional abilities for self-care (10). In Japan, the self-reliance support care model places greater emphasis on maintaining daily living skills (11). In response to this broader trend, China has also continued to promote a shift in health services from a disease-treatment-centered model toward a people-centered health approach in recent years. The *Healthy China 2030* Planning Outline emphasizes prevention, health promotion, and life-course health management, providing an important policy foundation for the development of proactive health (12). Building on this, the *14th Five-Year Plan for National Health* further advocates the concept of proactive health, emphasizing the need to move health management to earlier stages and enhance residents’ capacity for self-health management (13). Against this policy background, proactive health has gradually become an important approach to improving population health across the life course. In the field of older adult health, it emphasizes supporting older adults in adapting to physical and psychological changes, mobilizing available resources for health management, and maintaining a balance between functional changes and quality of life in daily living (14).

Proactive health shares some commonalities with international function-oriented care concepts, but their meanings and implementation pathways are not exactly the same. Western function-oriented care models mainly emphasize promoting individual independence by maintaining and using older adults’ remaining functional abilities (10), whereas proactive health in the Chinese context places greater emphasis on the collaborative participation of individuals, families, institutions, and social resources, which reflects the influence of collectivist values (15). Thus, although international experience can provide useful references for functional maintenance and independence support, it can not be directly transferred to Chinese LTCFs without considering the cultural and service-system context. Against this background, it is necessary to further examine the proactive health service needs of older adults with functional impairment in Chinese LTCFs. This makes it necessary to look first at what existing research in China has explored, and where its explanatory reach ends.

At present, research on proactive health among older adults with functional impairment in Chinese LTCFs mainly falls into three areas. The first area concerns proactive health-related behavioral capacity, with studies using scale-based tools to assess older adults’ level of proactive health and identify related influencing factors (16, 17). The second area focuses on smart older care service models, which use big data platforms to connect medical and long-term care resources and thereby improve the accuracy and continuity of services (18). The third area concerns the integration and evaluation of traditional Chinese medicine-based proactive health service systems, emphasizing the incorporation of the “preventive treatment of disease” concept, the development of service quality evaluation systems, and the establishment of health management models tailored to the needs of an aging population (19).

Although previous studies have made progress in the above three areas, research on the proactive health service needs of older adults with functional impairment in LTCFs remains limited. First, at the level of individual behavioral capacity, most existing studies have used quantitative surveys to describe proactive health-related abilities and identify associated influencing factors. However, quantitative methods may have limited capacity to capture older adults’ subjective experiences, cultural meanings, and complex contextual factors in daily institutional care. They may also be insufficient for exploring “hidden needs” and understanding the cultural and psychological mechanisms behind behaviors. This highlights the unique value of qualitative research in examining unmet proactive health service needs among older adults with functional impairment in LTCFs (16, 17). Second, in the Chinese context, the development of proactive health awareness and related behavioral decisions among older adults with functional impairment is shaped not only by family roles, care relationships, and institutional management practices, but also by macro factors, including supply–demand contradiction of long-term care human resources, uneven distribution of medical resources, and regional differences in the implementation of the *Healthy China 2030* policy (20, 21). However, existing studies have paid limited attention to how family, institutional, and macro factors shape the proactive health awareness, need expression, and behavioral choices of older adults with functional impairment in Chinese LTCFs.

Given these gaps, this study addresses the following research questions:

RQ1: In the context of Chinese LTCFs, what unmet proactive health service needs hinder older adults with functional impairment from shifting from passive receipt of care to active participation in health maintenance and functional preservation?

RQ2: How do service structures within LTCFs, traditional care beliefs, and family participation influence the proactive health awareness, health-related decision-making, and behavioral choices of older adults with functional impairment?

To address these questions, this study uses qualitative methods to explore the lived experiences of older adults with functional impairment and nursing staff regarding unmet needs in proactive health services. The findings may inform improvements in frontline care practices and provide a preliminary theoretical basis for developing person-centered service models in comparable LTCF contexts.

## 2 Methods

### 2.1 Study design

This study employed a descriptive qualitative design to explore the “unmet needs” of older adults with functional impairment and nursing staff in proactive health services in LTCFs. Descriptive qualitative research directly presents participants’ experiences and phenomena in language close to their own. It is therefore suitable for identifying unmet needs in complex situations (22). The design and reporting of this study followed the Standards for Reporting Qualitative Research (SRQR).

### 2.2 Setting and sample

A secondary integrated medical and older care institution affiliated with a tertiary Grade A hospital in Chongqing was selected as the study site. This institution is a national demonstration unit for integrated medical and older care and provides medical care, nursing care, long-term care, and rehabilitation services. It also has a streamlined referral pathway for residents who require further medical treatment. The institution has a relatively large activity area, regularly organizes various recreational activities, and offers both single and double rooms. Approximately 59% of its beds are used for older adults with functional impairment, which is broadly comparable to the national average of 67% in LTCFs. Therefore, this institution was considered relevant to the context of this study.

The study participants included older adults with functional impairment and nursing staff. The inclusion criteria for older adults with functional impairment were: (1) age ≥ 60 years; (2) residence in this LTCF for ≥ 6 months; (3) functional ability ranging from mild to severe impairment, assessed according to the *Specification for ability assessment of older adults* (GB/T 42195–2022) (23); (4) no diagnosed mental disorders; (5) no significant communication barriers; (6) provision of informed consent and voluntary participation. The exclusion criteria were inability to complete the interview due to illness or other personal reasons.

The inclusion criteria for nursing staff were: (1) working in this LTCF for ≥ 1 year; (2) primarily responsible for the care of older adults with functional impairment; and (3) provision of informed consent and voluntary participation. The exclusion criteria were those planning to leave within 3 months or those in non-direct care roles (such as administrative staff).

Sampling was conducted using a combination of purposive sampling and maximum variation sampling (24), ensuring diversity in gender, age, educational level, Degree of functional impairment (for older adult participants), as well as position and years of experience (for nursing staff). The sample size was determined based on data saturation, defined as the point at which no new themes emerged (25). After interviewing the 13th older adult participants and the nursing staff member, no new themes were identified. Two additional interviews were then conducted in each group to confirm saturation, with no new themes observed. Finally, 15 older adult participants and 13 nursing staff were included.

### 2.3 Researcher characteristics and reflexivity

All members of the research team had received training in qualitative research methods and were familiar with the principles of qualitative interviewing and data analysis. No prior personal relationship existed between the researchers and participants before recruitment. All members of the research team shared the same linguistic and cultural background as the participants, which facilitated communication and contextual understanding. At the same time, the researchers’ academic backgrounds in nursing and geriatric care may have shaped their assumptions about proactive health and institutional care. To reduce the influence of these understandings and assumptions on data interpretation, the research team actively engaged in reflexivity and discussion throughout data collection and analysis. One to two reflexive discussions were held each week. During data collection, the team reflected on thoughts, feelings, and emerging impressions from recent interviews, considered whether the interview questions or probing strategies needed adjustment, and discussed whether the researchers’ assumptions might have influenced participants’ responses. During data analysis, the discussions focused on the interpretation of ambiguous statements, the possible influence of the researchers’ nursing backgrounds and professional experience on coding decisions, and whether the emerging themes remained close to participants’ original accounts.

### 2.4 Data collection

Data were collected from July to September 2025 using semi-structured interviews. Based on a literature review (15, 26) and discussions within the research team, an initial interview guide was developed. Two older adults with functional impairment and two nursing staff who met the inclusion criteria were recruited for pilot interviews (pilot data were not included in the final analysis), and the guide was subsequently revised in consultation with a geriatric nursing expert and a head nurse specializing in geriatric care. The final interview guide is presented in Tables 1, 2.

**Table 1:** Semi-structured interview outline for older adults with functional impairment.

Semi-structured interview outline for older adults with functional impairment
Do you usually pay attention to your physical health, take part in activities that are beneficial to health, or actively seek health-related information? If so, what kinds of activities or information are involved?
What do you think prevents you from engaging in these health-promoting activities?
What measures in the long-term care facility make it easier for you to engage in these health-promoting activities?
If the long-term care facility were to organize activities in the future, what kinds of activities would you be willing to take part in and find helpful?
When your family members visit, do they talk with you about health-related matters? What kind of support would you like them to provide to help you take a more active role in managing your health?

**Table 2:** Semi-structured interview outline for nursing staff.

Semi-structured interview outline for nursing staff
In your opinion, among older adults with functional impairment under your care, what specific proactive health behaviors do you observe in daily life?
In your daily work, in what ways do you encourage or support older adults with functional impairment under your care to do things by themselves or take a more active role in their own health? What measures do you usually use to support them?
What kinds of knowledge or skills do you think older adults and nursing staff need to strengthen in order to support proactive health? What difficulties have you encountered in this process?
What resources do you think the long-term care facility currently provides to help older adults increase their engagement in healthy behaviors? What additional resources or support could be provided to better support this?
What role do family members play in supporting proactive health among older adults with functional impairment? What kinds of support do you think they can provide?

The first author conducted the interviews, while the co-first author recorded non-verbal cues and contextual notes during the interviews. Before each interview, participants were informed of the study purpose, procedures, and confidentiality arrangements, and written informed consent was obtained. The researchers also introduced themselves and explained their roles in the study to establish rapport. During the interviews, techniques such as confirmation and probing were used based on participants’ responses to ensure completeness of the data. Interviews were conducted in quiet and private rooms within the institution (such as activity rooms and meeting rooms), and peak care hours (8:00–10:00, 14:00–16:00) were avoided. Each interview lasted 15–50 min (mean = 19.5 min).

### 2.5 Data analysis

Within 24 h after each interview, two researchers transcribed the audio recordings verbatim into text, removing any identifying information. In cases of unclear or incomplete information during transcription, participants were contacted for clarification to ensure data accuracy. To enhance rigor, the transcriptions were cross-checked against the original audio recordings, and key information was confirmed with participants when necessary.

Data were managed using NVivo 20 and analyzed using inductive qualitative content analysis. The first author repeatedly read all transcripts to gain an overall understanding of the data. Words, sentences, or paragraphs related to the study aim were identified as meaning units, condensed according to their underlying meanings, and gradually developed into codes. An initial codebook was then developed, including code names, definitions, inclusion and exclusion criteria, and representative examples. The preliminary codebook contained 53 initial codes.

To improve the clarity and applicability of the codebook, the first author and the co-first author selected approximately 20% of the transcripts for coding, including both older adult and nursing staff interviews. Coding differences were discussed by returning to the original text, and the codebook was revised by merging overlapping codes, clarifying code definitions, and adding representative examples. After refinement, 49 formal codes were retained.

Using the revised codebook, the first author and the co-first author independently coded the full dataset. Intercoder agreement was calculated before consensus discussion using the identified meaning units as the unit of analysis, with Cohen’s kappa reaching 0.77. Disagreements were first discussed by the two coders. When consensus could not be reached, a third researcher was consulted. Finally, similar codes were grouped into sub-themes, and the main themes were developed through repeated comparison and discussion while continually returning to the original transcripts.

After the main themes were identified, we also reviewed whether unmet needs of proactive health service showed notable variations across participant characteristics, including age, educational level, length of stay, and functional status.

### 2.6 Rigor

Lincoln and Guba’s criteria of credibility, dependability, confirmability, and transferability were used to ensure the rigor of this study (27).

Credibility was enhanced through recording of all interviews, verbatim transcription, checking transcripts against the original recordings, and confirming unclear or key information with participants when necessary. During data analysis, preliminary codes, sub-themes, and themes were discussed within the research team. Representative quotations were also used to support the interpretation of the findings.

Dependability was supported by a systematic and transparent analytic process. Data were managed using NVivo 20 and analyzed using inductive qualitative content analysis. A preliminary codebook was developed and refined. Two researchers independently coded the full dataset using the revised codebook, and intercoder agreement was calculated before consensus discussion. Coding disagreement was first discussed by the two coders with reference to the original transcripts. When consensus could not be reached, a third researcher was consulted.

Confirmability was enhanced through reflexive discussions, analytic memos, and an audit trail. The audit trail included anonymized transcripts, coding notes, different versions of the codebook, records of disagreement resolution, and records of theme development. These procedures helped ensure that the findings were grounded in the data rather than shaped mainly by the researchers’ assumptions.

Transferability was supported by providing detailed descriptions of the research setting, participant characteristics, sampling strategies, data collection procedures, and analytic process. These details allow readers to assess whether the findings may be applicable to other LTCF contexts.

### 2.7 Ethical considerations

Ethical approval for this study was granted by the Ethics Committee of the First Affiliated Hospital of Chongqing Medical University (Approval No. 2023–035). All participants provided informed consent before participation. Data confidentiality and security were strictly maintained throughout data collection and analysis, with access restricted to the research team. The study was conducted in accordance with the Declaration of Helsinki.

## 3 Results

### 3.1 Participant characteristics

This study interviewed 15 older adults with functional impairment and 13 nursing staff. The average age of older adults with functional impairment was 86.13 years (ranging from 71 to 95 years), with an average length of stay of 4 years (ranging from 1 to 7 years). The average age of nursing staff was 38.50 years (ranging from 29 to 51 years), and the average working duration was 10.58 years (ranging from 1 to 26 years). The general information of the participants is listed in Tables 3, 4.

**Table 3:** Characteristics of older adults with functional impairment.

No.	Gender	Age	Education level	Degree of functional impairment	Length of stay
R1	Male	80s	Junior college and above	Severe	>3 yr
R2	Female	90s	Junior college and above	Moderate	≤3 yr
R3	Female	70s	Primary school	Moderate	≤3 yr
R4	Female	80s	Junior college and above	Moderate	>3 yr
R5	Male	80s	Junior college and above	Moderate	≤3 yr
R6	Female	90s	Primary school	Moderate	≤3 yr
R7	Male	90s	High school/ secondary school/ technical school	Moderate	>3 yr
R8	Female	80s	High school/ secondary school/ technical school	Moderate	≤3 yr
R9	Female	90s	Junior college and above	Mild	≤3 yr
R10	Female	80s	Junior college and above	Moderate	>3 yr
R11	Female	90s	High school/ secondary school/ technical school	Mild	>3 yr
R12	Male	90s	Junior college and above	Moderate	>3 yr
R13	Female	70s	High school/ secondary school/ technical school	Severe	≤3 yr
R14	Female	70s	High school/ secondary school/ technical school	Severe	≤3 yr
R15	Female	80s	Junior college and above	Mild	>3 yr

**Table 4:** Characteristics of nursing staff.

No.	Gender	Age	Education level	Position	Working years in the LTCF
S1	Male	20s	Junior college and above	Nurse	6 yr
S2	Female	30s	Junior college and above	Care Specialist	6 yr
S3	Female	40s	Junior college and above	Nurse	26 yr
S4	Female	20s	Junior college and above	Care Assistant	1 yr
S5	Female	30s	Junior college and above	Nurse	15 yr
S6	Female	50s	High school/ secondary school/ technical school	Care Assistant	7 yr
S7	Female	40s	Junior college and above	Nurse	20 yr
S8	Female	50s	High school/ secondary school/ technical school	Care Assistant	8 yr
S9	Female	30s	Junior college and above	Care Specialist	10 yr
S10	Female	20s	Junior college and above	Nurse	8 yr
S11	Female	40s	High school/ secondary school/ technical school	Care Assistant	6 yr
S12	Female	30s	Junior college and above	Nurse	14 yr
S13	Female	40s	High school/ secondary school/ technical school	Care Assistant	5 yr

### 3.2 Identified themes

**Figure 1:**
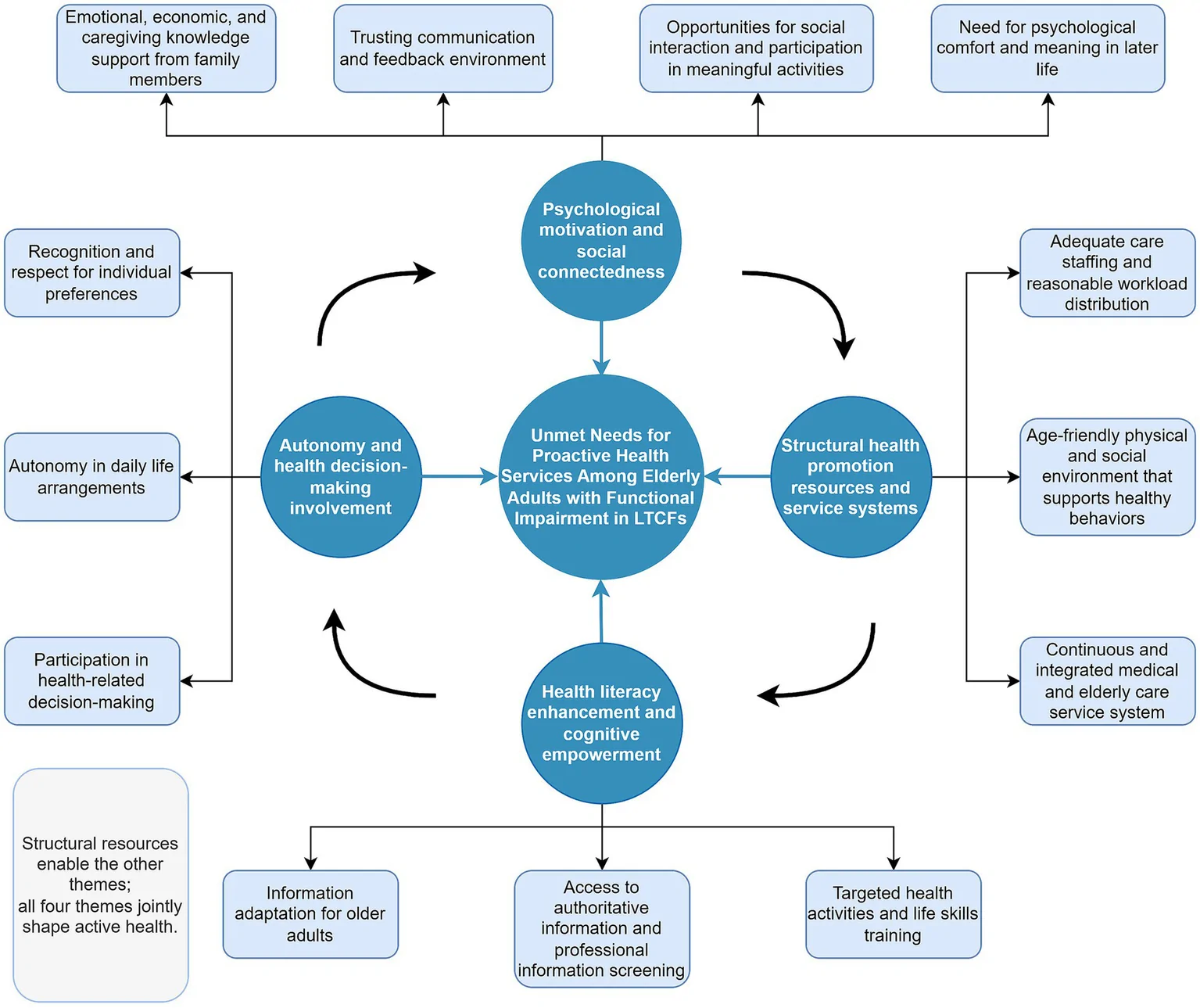
Four main themes and sub-themes of unmet needs in proactive health services among older adults with functional impairment in LTCFs. LTCFs, long-term care facilities.

Based on the interview data, four main themes were identified: autonomy and health decision-making involvement, health literacy enhancement and cognitive empowerment, psychological motivation and social connectedness, and structural health promotion resources and service systems (Figure 1).

#### 3.2.1 Autonomy and health decision-making involvement

In the context of care in LTCFs, autonomy is an important foundation for older adults with functional impairment to participate in proactive health practice. The interviews showed unmet needs related to autonomy in three areas: recognition and respect for individual preferences, autonomy in daily life arrangements, and participation in health-related decision-making.

##### 3.2.1.1 Sub-theme 1: recognition and respect for individual preferences

The subjective needs of some older adults with functional impairment are not promptly identified or adequately addressed. Their individual feelings are often misunderstood, ignored, or marginalized.

One older adult with severe functional impairment described an experience of being misunderstood:

*“I once thought about dying. Before, they would leave the cold air on all night in my room, giving me terrible leg cramps and headaches. But then the director came to visit us that day, and after that, the nursing staff made some changes.”* (R14).

A nursing staff member described the same situation from a care perspective:

*“It’s really hot now. Her (R14) roommate need to turn on the air conditioner, so we have to turn it on. We set it to a comfortable temperature, but she insists on turning it off. But I do not understand why she did not feel cold in the hall, even with the air conditioner on.”* (S8).

Unmet needs for autonomy were also reflected in major life decisions, such as entering an LTCF. Some residents’ preferences were overridden by family concerns about safety, and they eventually complied with family arrangements.

*“Whether I’m happy or not, nothing will change. Once they send you here, there’s no going back. I did not want to live here. My grandson brought me here because he was worried about leaving me all alone after my husband passed away.”* (R6).

##### 3.2.1.2 Sub-theme 2: autonomy in daily life arrangements

Under the unified management model of LTCFs, older adults with functional impairment had limited control over some aspects of their daily lives. This was especially evident in meal provision. Standardized meals did not always meet individual needs, leaving some older adults in a passive position.

*“When I’m hungry, I eat a bit more, when I’m not, I eat less. The meals… sometimes they are okay, sometimes the rice is really hard, and sometimes the food is cold. I just eat what I can. What else can I do?”* (R6).

Access to activity information and participation in group activities are also constrained. Some information is not fully communicated to older adults with functional impairment, while staff sometimes pre-selected participants based on physical condition.

*“I like activities the most. I would go there myself, even if it’s difficult to move. But lately there have not been any activities, and no one has told me.”* (R3).

*“Some older adults have difficulty moving, so some activities aren’t really suitable for them.”* (S2).

##### 3.2.1.3 Sub-theme 3: participation in health-related decision-making

In health management decisions, older adults with functional impairment had limited opportunities to receive information, express their views, and participate in decisions related to their own health.

*“When I first came here, my main problem was heart disease. My daughter contacted the doctor, and they told me I needed to take medication. They said I was anemic, but I just could not get used to it. It was mostly milk and condensed milk—too sweet, really sickly.”* (R9).

Similar issues were also seen in nutritional care. Although professionals were available, related services were often delivered in a one-way manner, with limited explanation or feedback, making it difficult for older adults with functional impairment to understand and engage with these services.

*“There is a nutritionist, but they never explain things clearly. Why are the meals arranged this way? What are they based on? And how are we supposed to actually put these into practice in our meals?”* (R1).

#### 3.2.2 Health literacy enhancement and cognitive empowerment

Older adults with functional impairment can participate more actively in health management only when they are able to obtain, understand, and use health information. However, the interviews showed unmet needs in access to understandable information, authoritative information screening, and targeted guidance.

##### 3.2.2.1 Sub-theme 1: information adaptation for older adults

The main ways for older adults with functional impairment to obtain health knowledge include television, newspapers, and health education provided by LTCFs. However, visual impairment, memory decline, and limited digital skills made it difficult for them to access and use this information.

*“I mostly get health information from the TV and lecture, but my eyesight is not good, so I cannot really see it. I just listen for a while, but I do not remember much either.”* (R8).

At the same time, some older adults with functional impairment are not able to use smart devices proficiently. This digital divide further limits their access to health information.

*“Some of them cannot use smartphones, so their sources of health information are much more limited. A lot of information is now released online, while printed materials are updated very slowly.”* (S7).

##### 3.2.2.2 Sub-theme 2: access to professional information

In the context of increasingly complex information sources, older adults with functional impairment needed reliable and professionally filtered information. However, they often lacked the knowledge and guidance needed to judge the reliability of health information.

*“You really have to watch out for those health supplements, there are traps everywhere. Without someone to advise you, your own thinking is too limited, and sometimes you still end up getting fooled.”* (R1).

Nursing staff also mentioned that what older adults with functional impairment need is not simply more information, but information that has been professionally filtered, is authoritative, and matches their way of understanding.

*“AI is very popular now. If institutions had a robot specifically for older adults, and the health education it provided were authoritative—say, based on guidelines or expert consensus—that would be very important. It would help them filter that information.”* (S12).

##### 3.2.2.3 Sub-theme 3: targeted health activities and life skills training

The interviews showed that existing activities and guidance in LTCFs are often overly simple and homogeneous, making it difficult to meet the differentiated needs of older adults with different functional abilities. As a result, some of them become less willing to participate.

*“I did not go to the group exercise activity. I felt it was too little exercise. I might as well just go for a walk on my own.”* (R12).

*“The dance they have been teaching does not seem very well prepared. Older adults’ abilities differ and what suits one person may not suit another. They could be a bit more professional in how they run the activity.”* (R15).

Current services also focused mainly on physical exercise, with less attention to practical skills needed in an information-based society. Those with memory decline especially needed repeated, face-to-face guidance in using smart devices.

*“Actually, because of illness, we forget things very quickly. We can suddenly get confused about how to send a text message, and smartphones are even harder for us to figure out. It would really help if someone could teach us how to use a mobile phone face to face, for example how to make video calls.”* (R4).

#### 3.2.3 Psychological motivation and social connectedness

The motivation of older adults with functional impairment to engage in health management was shaped not only by their own attitudes, but also by family support, communication, social relationships, and psychological comfort. The interviews showed unmet needs in family involvement, safe expression, meaningful interaction, and continuous emotional support.

##### 3.2.3.1 Sub-theme 1: emotional, economic, and caregiving knowledge support from family members

After entering the LTCF, older adults with functional impairment still need family support, including emotional connection, replenishment of daily supplies, and coordination in health care. The interviews showed that these needs were not fully met in practice.

*“It would be great if they gave me a call. I do not know how to make phone calls myself. That’s good enough for me. They’re too far away, and they do not have time to come over.”* (R3).

Although some older adults with functional impairment had emotional needs, they often suppressed their own expression because they did not want to burden their adult children.

*“We’re old now, and it’s not easy for us to go out anymore. It affects everyone’s work and daily life, and we feel bad about it ourselves. We do not want to burden them.”* (R4).

Nursing staff noted that family support in daily supplies and illness-related care sometimes declined over time.

*“For some older adults and at first the family would still buy some online. But now some family members hardly bother anymore. Some of these older adults barely even have enough clothes to wear now.”* (S6).

*“Family members also need to understand illnesses such as cognitive impairment, Parkinson’s disease, and diabetes. Once they understand the condition, we do not have to keep explaining things over and over. For example, sometimes the person clearly has diabetes, and the family still brings grapes every time.”* (S13).

##### 3.2.3.2 Sub-theme 2: trusting communication and feedback environment

Smooth communication channels and a supportive feedback environment are important for older adults with functional impairment and nursing staff. However, the interviews suggested that this need was not adequately met. They often hesitate to express their true opinions and needs.

*“We have all been here for a long time, and everyone knows each other. That makes some things hard to bring up.”* (R1).

Interviews with nursing staff also suggested that a trusting and responsive communication environment has not yet taken shape.

*“Sometimes older adults do not trust us nursing staff. Sometimes family members do not understand us either, and that makes things very difficult. They do not support our work.”* (S6).

##### 3.2.3.3 Sub-theme 3: opportunities for social interaction and participation in meaningful activities

Although LTCFs provide a shared living space, the needs of older adults with functional impairment for deeper social connection were not naturally met. Interactions often remained superficial.

*“We are not really familiar with the other people here, and it is impossible to have heart-to-heart conversations with them. You may know many people, but how many truly understand you?”* (R1).

*“There is not much chatting together. We all came from outside and do not really know each other well.”* (R7).

Some older adults with functional impairment who still wanted to engage socially, had to reduce their social activities because they could not find companions with similar interests.

*“I like learning characters, writing, and playing mahjong, but there is no one to do them with me.”* (R3).

##### 3.2.3.4 Sub-theme 4: need for psychological comfort and meaning in later life

Some older adults of advanced age or with severe functional impairment expressed weariness toward life and low motivation to engage in health-related learning, suggesting unmet needs for emotional comfort and ongoing psychological support.

*“I do not really care about my health. I just want to go soon … I’m already over ninety. I feel I’ve lived long enough. I do not want to learn anything.”* (R11).

#### 3.2.4 Structural health promotion resources and service systems

Proactive health practice among older adults with functional impairment depends not only on their own participation and the cooperation of nursing staff, but also on the support of structural resource allocation and service systems. The interviews showed unmet needs in care staffing, age-friendly environmental support, and continuous integrated medical and older adult care services.

##### 3.2.4.1 Sub-theme 1: adequate care staffing

Staffing shortages limited the capacity of LTCFs to provide services beyond basic care. Under heavy workloads, nursing staff often had less time for activities that supported residents’ physical function and social vitality.

*“We used to have quite a lot of activities, but now there are fewer staff, so we just do not have the time for them anymore, and we cannot play together with older adults the way we used to.*” (S11).

*“Now there are more old adults coming, but there are still too few staff. To be honest, they work really hard.”* (R2).

##### 3.2.4.2 Sub-theme 2: age-friendly physical environment that supports healthy behaviors

LTCFs are the main living spaces for older adults with functional impairment. The age-friendliness of the environment directly affects their mobility, activity participation, and daily life. The interviews suggested that spatial layout, activity space and functional support equipment did not fully meet their needs.

*“No one pushes our wheelchairs to take us out into the sun. There’s a slope on the way to the little garden, and it’s too steep for me, and we cannot go by ourselves.”* (R8).

*“Sometimes they held activities on the second floor. There were lots of people in wheelchairs and on crutches, and there was nowhere to put my wheelchair. Even going to the toilet was difficult.”* (R7).

Nursing staff also noted that some institutions had more age-friendly facilities.

*“Some geriatric hospitals are better equipped with age-friendly facilities. The dining tables, beds, and toilet seats were all designed with older adults in mind.”* (S10).

##### 3.2.4.3 Sub-theme 3: continuous and integrated medical and older care service system

Some older adults with functional impairment perceived a gap between the institution’s integrated medical and older adult care positioning and the actual medical services they could access, which did not fully meet their expectations.

*“The nurse explained to me that this is an LTCF, not a hospital. But since this place is called an integrated medical and older adult care institution, they still need to pay attention to health problems. As for some of the treatment-related issues I mentioned, I feel the doctors here do not pay enough attention to them.”* (R9).

*The coordination between institutional care and external specialty care remains insufficient, making it harder for older adults with functional impairment to access specialist services when needed.*

*“Although living in this integrated medical and older adult care* institution*, sometimes seeing a specialist is really troublesome. You have to go to the hospital, and it is very inconvenient. It also affects my family members’ work.”* (R4).

## 4 Discussion

In recent years, proactive health has become increasingly prominent in China’s aging policy framework. Its core emphasis is a shift from disease treatment alone to lifelong health management and functional maintenance. As major providers of long-term care, LTCFs are important settings for translating this shift into daily care practice. However, for older adults with functional impairment, proactive health practice does not depend solely on their own willingness or participation; it also requires continuous support from LTCFs and families. When such support is insufficient, older adults may find it difficult to access health information, maintain motivation, or engage in health-promoting behaviors in daily institutional life. Therefore, understanding their unmet needs in proactive health services is important for improving quality of life and for supporting the development of proactive health services in LTCFs.

This study examined existing gaps in services promoting proactive health behaviors from four dimensions: autonomy and health decision-making involvement, health literacy enhancement and cognitive empowerment, psychological motivation and social connectedness, and structural health promotion resources and service systems. The findings showed that although policies emphasize that individuals are primarily responsible for their own health, in reality, for older adults with functional impairment, their proactive health behaviors are still constrained by various factors.

Older adults with functional impairment in LTCFs often face a loss of autonomy. From the perspective of proactive health, autonomy is not only related to dignity, but also to their participation in health management as active agents rather than passive care recipients (8). This study found that their subjective feelings, daily life arrangements, and health-related decisions often did not receive adequate response within the institution, which is consistent with previous studies (8, 28, 29). When older adults’ experiences and wishes are not sufficiently valued, their sense of control over daily life and health management may be weakened, and care services may become less responsive to their actual needs. This may further limit their willingness and ability to participate in proactive health management (30). This problem is closely related to the severity of functional impairment, imbalances in the staff-to-resident ratio, paternalistic tendencies in care, and institutional arrangements in LTCFs (31–33).

It is worth noting that some nursing staff used the term “old children” in the interviews. In Chinese, this term carries an affectionate tone, it may unintentionally reflect a paternalistic framing of older adults with functional impairment, making substitute decision-making or coaxing appear acceptable and weakening their autonomy as adults. At the same time, adult children’s tendency to take over everything out of concern for their parents’ safety may further aggravate the loss of autonomy in this population (34).

To address the weakening of autonomy, LTCFs could preserve daily life choices for older adults with functional impairment as far as possible, including choices related to meals, daily routines, and activity arrangements. Shared decision-making could also be adopted in health-related and other major decisions, with respect for their right to express their views (29, 35, 36). In addition, continuous training, teamwork, increased staffing, and the promotion of empowering care may help improve nursing staff’s skills, reduce work pressure, and discourage doing things on behalf of older adults with functional impairment (8, 37).

Autonomy alone is not sufficient to enable proactive health behaviors. Older adults with functional impairment also need the knowledge and skills to understand health risks, judge the reliability of health information, and develop awareness of prevention and self-management. From the perspective of proactive health, insufficient health literacy may prevent them from translating their willingness to pursue health into appropriate health actions, making them more likely to respond passively after health problems occur (16). It may also increase their vulnerability to misleading health information and related fraud (38–40). This study found that they faced multiple barriers in obtaining and understanding health information, including declines in vision and hearing, memory loss, the digital divide, and a lack of individualized information services. These barriers make it difficult for them to meet their health knowledge needs, which is consistent with previous studies (26, 41, 42).

Among these barriers, declines in vision and hearing were frequently reported. Against this background, LTCFs may need to pay greater attention to the age-friendly adaptation of health information. Health education could focus on key messages and use multimodal approaches, such as large-print materials, diagrams, and hands-on demonstrations. These approaches may help support understanding and memory. The teach-back method may also be used to assess whether older adults with functional impairment have truly understood the information. In addition, previous studies have shown that interactive group education combined with individualized guidance is a more acceptable and effective form of health education for them (43, 44).

Proactive health practice also requires psychological motivation and social connectedness. From the perspective of proactive health, social support may help older adults with functional impairment maintain psychological resilience and health empowerment, thereby strengthening their willingness to participate in health management (45). In this study, older adults with functional impairment showed unmet needs for psychological and social support, which is consistent with previous research (46). The interviews suggested that, after moving into LTCFs, older adults with functional impairment often needed to rebuild emotional connections in a new environment because their original social networks had been disrupted. However, within the institution, deep social bonds may be difficult to form among older adults, and mutual trust between older adults with functional impairment and nursing staff may not always be fully established (47). As a result, emotional support within the institution may remain limited. In the Chinese cultural context, family remains an important source of emotional support for older adults with functional impairment (48). However, in our interviews, interactions with family members appeared to be largely limited to material support and remote contact (49). At the same time, some older adults with functional impairment reduced contact with their adult children on their own initiative because they did not want to become a burden. This may further leave their emotional needs unmet (50). Such unmet emotional needs, together with the guilt associated with functional impairment, may further affect their health status (51).

Therefore, LTCFs could strengthen social ties and emotional support for older adults with functional impairment at both the family and institutional levels. At the family level, institutions can strengthen emotional connection by regularly organizing family meetings, helping older adults with functional impairment use communication technologies, and establishing family liaison roles (52). At the institutional level, interest-based groups may be established to help older adults with functional impairment build stable peer relationships. Intergenerational group reminiscence activities and life review therapy may also be introduced. Older adults with functional impairment may be encouraged to take on roles such as activity organizers or volunteers, where feasible, in order to enhance their sense of self-worth and reduce feelings of weariness toward life. Institutions could also strengthen daily emotional communication with older adults. Mutual trust can be improved by enhancing nursing staff’s professional skills and communication strategies (53). Meanwhile, effective feedback mechanisms could be established so that both sides can express themselves openly.

Proactive health practice is difficult to sustain through individual willingness alone; it also depends on whether LTCFs can provide the structural conditions needed to translate health intentions into daily practice. This study found that the structural resource needs of older adults with functional impairment and nursing staff were mainly concentrated in human resources, the environment, and medical services (26). From the perspective of proactive health, these deficiencies may limit their opportunities for rehabilitation, social participation, and continuous health management, and may also weaken their motivation to engage in health-promoting activities (54, 55).

These structural resource gaps should be understood within China’s broader long-term care policy and service context. National policies have increasingly emphasized improving care capacity, supporting workforce development, building age-friendly environments, and promoting integrated medical and older adult care services (56). However, translating these goals into everyday institutional practice remains challenging. First, the shortage of professional care workers and differences in their competencies limit the ability of LTCFs to provide services beyond basic care. Relevant data show that China requires at least 13 million care workers to meet the needs of older adults with complete or partial functional impairment, whereas the current workforce is only about 1 million. Among them, the proportion of certified care workers is below 32% (20). This may explain why nursing staff in this study often had to prioritize routine care over rehabilitation support, activity organization, and individualized health guidance. Within available resources, LTCFs may consider introducing selected digital health monitoring devices or assistive technologies to help reduce the care burden (57, 58).

Second, age-friendly environmental modification remains difficult at the institutional level. Some LTCFs have been adapted from existing older adult care institutions, while others have been converted from ordinary buildings. Funding constraints, original building structure, limited space, and geographical conditions may all restrict environmental modification (59). The study site is located on a steep slope, which also reflects how existing environmental conditions may limit barrier-free mobility and participation in activities. Where feasible, LTCFs could adapt existing resources, optimize accessible pathways, improve activity spaces, and introduce age-friendly facilities to reduce environmental barriers to proactive health behaviors (60).

Third, integrated medical and older adult care services still vary in continuity and coordination. Although integrated care has become an important direction in China, institutions differ in the extent to which medical resources are embedded and in their service capacity. Institutions may rely on one or more approaches, such as on-site clinics, visiting medical services, or green referral channels, but their coverage capacity varies depending on institutional resources. Some institutions can largely meet the medical and health management needs of older adults with functional impairment, whereas others still have clear gaps (61). Thus, even in an integrated medical and older adult care institution, older adults with functional impairment may still need external hospitals for specialist care, complex chronic disease management, or further diagnosis and treatment. LTCFs may therefore consider optimizing internal ward rounds, chronic disease follow-up, and convenient service pathways such as online follow-up consultations to improve continuity and accessibility of care.

In addition, a brief subgroup comparison suggested that unmet needs were not expressed in exactly the same way across participants. The most noticeable variation appeared to be related to length of stay: older adults with functional impairment who had stayed for a shorter time tended to need more family contact to help them adapt and feel emotionally secure. In contrast, older adults with functional impairment who had stayed for a longer time were more likely to talk about psychological comfort, loneliness, and the need for meaningful activities. Some differences in emphasis were also observed in relation to functional status, although these differences were less consistent. These observations should be interpreted cautiously, as they reflect qualitative variations in participants’ experiences rather than formal comparisons between statistically comparable subgroups.

In conclusion, proactive health among older adults with functional impairment in LTCFs depends not only on individual capacity, but also on support at multiple levels. Viewed through the lens of unmet needs, promoting proactive health is not simply a matter of asking them to take the initiative, but of providing conditions that are accessible, understandable, and dependable. These findings suggest that, within available resources, LTCFs could support proactive health by strengthening autonomy, empowerment, social connection, and structural support in daily care practice. The transferability of these findings should be interpreted with caution. This study was conducted in a single hospital-affiliated integrated medical and older adult care demonstration institution in Chongqing, which may have better medical resources, staffing support, and service infrastructure than smaller private LTCFs, rural facilities, or institutions in less economically developed regions. Therefore, the findings should not be directly generalized to all LTCF settings in China, but may provide context-specific insights for comparable institutions.

## 5 Limitations

This study has several limitations. First, the sample size was relatively small, and participants were recruited from only one LTCF in Chongqing. The findings may be shaped by the specific regional, institutional, staffing, and sociocultural context of the study site. Therefore, they should be interpreted with caution and may not be directly transferable to LTCFs with different resource conditions, service models, resident characteristics, or local care cultures. Future multicenter studies involving diverse regions and institution types are needed to further examine the applicability of these findings. Second, family members were not included because of their low level of participation. As family members are often involved in decision-making, emotional support, and care coordination, their absence may have limited our understanding of family involvement and the barriers families face in supporting proactive health. Future studies could incorporate the perspectives of older adults with functional impairment, nursing staff, and family members. Third, this study used a cross-sectional design and could not capture the dynamic changes in proactive health needs over time. The proactive health needs of older adults with functional impairment may change as their functional status changes or as institutional services evolve. Future longitudinal studies could further explore how these needs change throughout the course of institutionalization.

## 6 Conclusion

This qualitative study identified several unmet needs regarding proactive health promotion services among older adults with functional impairment in a single LTCF. The findings suggest that promoting proactive health practice in institutional settings requires not only strengthening older adults’ own health-related capacities, but also building a supportive environment in terms of care philosophy, health information provision, psychosocial support, and resource allocation.

Specifically, this study indicates that empowerment pathways may be explored at the individual, institutional, and societal levels. At the individual level, a shared understanding of proactive health among older adults with functional impairment, family members, and nursing staff could be promoted. Through shared decision-making, individualized health education, and function-maintenance training, older adults with functional impairment may be supported in retaining choices and opportunities for participation. At the institutional level, LTCFs could strengthen empowerment-oriented care training, optimize health education and daily activity programs, promote age-friendly environmental modification, increase care staffing, and improve visiting medical services and referral coordination. At the societal level, the findings may provide some reference for improving the long-term care service system. Future efforts may consider incorporating health education participation, function-maintenance support, shared decision-making, psychosocial support, and integrated medical and older adult care coordination into institutional service quality evaluation, while further strengthening workforce development, age-friendly environment construction, and the coordination of medical and older adult care resources.

Therefore, promoting proactive health practice among older adults with functional impairment may require a shift in care models from meeting basic needs toward an older-adult-centered approach oriented toward function maintenance and health participation. It should be noted that the above suggestions are based on the context of this study and should mainly be understood as practice implications for similar institutions. Their applicability still needs to be further examined across different regions and institutional settings.

## Data Availability

The original contributions presented in the study are included in the article/supplementary material, further inquiries can be directed to the corresponding author.
